# Supplementary material for: Multi‐dataset identification of innovative feature genes and molecular mechanisms in keratoconus
Source: J Cell Mol Med. 2024 Sep 19;28(18):e70079. doi: 10.1111/jcmm.70079 (PMC11412914; doi:10.1111/jcmm.70079)
Supplement: Supplementary file 2 — Material S2. [file JCMM-28-e70079-s002.zip › 7.GSEA analysis/4.MYRF/KEGG_NEUROTROPHIN_SIGNALING_PATHWAY.html]

Details for gene set KEGG\_NEUROTROPHIN\_SIGNALING\_PATHWAY[GSEA]

|  || Dataset | MYRF.group.cls#h\_versus\_l |
| Phenotype | group.cls#h\_versus\_l |
| Upregulated in class | l |
| GeneSet | KEGG\_NEUROTROPHIN\_SIGNALING\_PATHWAY |
| Enrichment Score (ES) | -0.3343029 |
| Normalized Enrichment Score (NES) | -1.6130315 |
| Nominal p-value | 0.026804123 |
| FDR q-value | 0.2278279 |
| FWER p-Value | 0.579 |
Table: GSEA Results Summary

  

Fig 1: Enrichment plot: KEGG\_NEUROTROPHIN\_SIGNALING\_PATHWAY      
 Profile of the Running ES Score & Positions of GeneSet Members on the Rank Ordered List

  

| SYMBOL | TITLE | RANK IN GENE LIST | RANK METRIC SCORE | RUNNING ES | CORE ENRICHMENT || 1 | CAMK2B | na | 283 | 0.659 | 0.0303 | No |
| 2 | PIK3R5 | na | 661 | 0.528 | 0.0503 | No |
| 3 | IRAK3 | na | 3091 | 0.291 | -0.0029 | No |
| 4 | CAMK2A | na | 4292 | 0.245 | -0.0233 | No |
| 5 | RPS6KA2 | na | 4975 | 0.223 | -0.0299 | No |
| 6 | NTRK1 | na | 5375 | 0.211 | -0.0291 | No |
| 7 | CALML6 | na | 5567 | 0.206 | -0.0226 | No |
| 8 | SOS1 | na | 6706 | 0.172 | -0.0454 | No |
| 9 | IRS4 | na | 7035 | 0.163 | -0.0454 | No |
| 10 | PIK3R1 | na | 7105 | 0.161 | -0.0380 | No |
| 11 | CALML5 | na | 7292 | 0.156 | -0.0342 | No |
| 12 | NGFR | na | 7411 | 0.153 | -0.0287 | No |
| 13 | BRAF | na | 8198 | 0.133 | -0.0436 | No |
| 14 | MAPK9 | na | 8409 | 0.129 | -0.0421 | No |
| 15 | MAPK12 | na | 8572 | 0.125 | -0.0395 | No |
| 16 | RPS6KA6 | na | 8598 | 0.125 | -0.0329 | No |
| 17 | KRAS | na | 9371 | 0.108 | -0.0489 | No |
| 18 | SORT1 | na | 9616 | 0.103 | -0.0499 | No |
| 19 | PIK3CG | na | 9794 | 0.100 | -0.0492 | No |
| 20 | IRAK1 | na | 10013 | 0.096 | -0.0499 | No |
| 21 | AKT2 | na | 10210 | 0.092 | -0.0502 | No |
| 22 | MAPK11 | na | 10606 | 0.085 | -0.0567 | No |
| 23 | PRDM4 | na | 10757 | 0.082 | -0.0562 | No |
| 24 | MAPK13 | na | 10951 | 0.079 | -0.0572 | No |
| 25 | CAMK2G | na | 10986 | 0.078 | -0.0536 | No |
| 26 | PSEN1 | na | 11022 | 0.078 | -0.0500 | No |
| 27 | PDPK1 | na | 11113 | 0.076 | -0.0482 | No |
| 28 | PIK3CA | na | 11255 | 0.074 | -0.0479 | No |
| 29 | SHC3 | na | 11433 | 0.071 | -0.0488 | No |
| 30 | PTPN11 | na | 11569 | 0.069 | -0.0487 | No |
| 31 | CALM2 | na | 11947 | 0.064 | -0.0558 | No |
| 32 | MAPK14 | na | 11965 | 0.064 | -0.0526 | No |
| 33 | NTRK3 | na | 12184 | 0.061 | -0.0553 | No |
| 34 | MAP3K3 | na | 12309 | 0.059 | -0.0555 | No |
| 35 | RPS6KA3 | na | 12372 | 0.058 | -0.0538 | No |
| 36 | MAPK10 | na | 12440 | 0.057 | -0.0524 | No |
| 37 | PIK3R2 | na | 12535 | 0.056 | -0.0519 | No |
| 38 | SOS2 | na | 12783 | 0.053 | -0.0559 | No |
| 39 | PLCG1 | na | 12816 | 0.052 | -0.0538 | No |
| 40 | TP53 | na | 12977 | 0.051 | -0.0554 | No |
| 41 | PLCG2 | na | 12987 | 0.050 | -0.0528 | No |
| 42 | IKBKB | na | 12994 | 0.050 | -0.0500 | No |
| 43 | YWHAB | na | 13423 | 0.045 | -0.0597 | No |
| 44 | CSK | na | 13727 | 0.041 | -0.0661 | No |
| 45 | MAP3K1 | na | 13876 | 0.039 | -0.0681 | No |
| 46 | KIDINS220 | na | 13931 | 0.039 | -0.0674 | No |
| 47 | MAP2K2 | na | 14012 | 0.038 | -0.0675 | No |
| 48 | RPS6KA5 | na | 14158 | 0.036 | -0.0695 | No |
| 49 | MAPK3 | na | 14382 | 0.034 | -0.0740 | No |
| 50 | FRS2 | na | 14920 | 0.029 | -0.0879 | No |
| 51 | RPS6KA4 | na | 15544 | 0.022 | -0.1046 | No |
| 52 | MAPK7 | na | 15567 | 0.022 | -0.1039 | No |
| 53 | FASLG | na | 16299 | 0.015 | -0.1242 | No |
| 54 | YWHAZ | na | 16350 | 0.014 | -0.1248 | No |
| 55 | TP73 | na | 16394 | 0.014 | -0.1252 | No |
| 56 | JUN | na | 16671 | 0.011 | -0.1325 | No |
| 57 | YWHAE | na | 16676 | 0.011 | -0.1320 | No |
| 58 | CALML3 | na | 17129 | 0.008 | -0.1446 | No |
| 59 | PIK3CB | na | 17199 | 0.007 | -0.1462 | No |
| 60 | RAP1A | na | 17348 | 0.006 | -0.1502 | No |
| 61 | GAB1 | na | 17603 | 0.003 | -0.1573 | No |
| 62 | CALM1 | na | 17693 | 0.002 | -0.1597 | No |
| 63 | ZNF274 | na | 17740 | 0.002 | -0.1610 | No |
| 64 | BAX | na | 17915 | 0.000 | -0.1660 | No |
| 65 | AKT1 | na | 18101 | -0.001 | -0.1712 | No |
| 66 | MAPK8 | na | 18612 | -0.006 | -0.1856 | No |
| 67 | RHOA | na | 18827 | -0.008 | -0.1913 | No |
| 68 | RAP1B | na | 18871 | -0.009 | -0.1920 | No |
| 69 | MAP2K7 | na | 19405 | -0.015 | -0.2065 | No |
| 70 | GSK3B | na | 19466 | -0.016 | -0.2074 | No |
| 71 | SH2B2 | na | 19706 | -0.018 | -0.2132 | No |
| 72 | MAPK1 | na | 20406 | -0.027 | -0.2318 | No |
| 73 | PIK3CD | na | 20433 | -0.027 | -0.2310 | No |
| 74 | PIK3R3 | na | 20464 | -0.027 | -0.2303 | No |
| 75 | IRAK4 | na | 20511 | -0.028 | -0.2300 | No |
| 76 | PRKCD | na | 20569 | -0.028 | -0.2300 | No |
| 77 | YWHAG | na | 20695 | -0.030 | -0.2319 | No |
| 78 | MAP2K5 | na | 21130 | -0.035 | -0.2424 | No |
| 79 | ATF4 | na | 21458 | -0.039 | -0.2495 | No |
| 80 | FOXO3 | na | 21594 | -0.041 | -0.2511 | No |
| 81 | RAF1 | na | 21720 | -0.042 | -0.2522 | No |
| 82 | HRAS | na | 21856 | -0.044 | -0.2535 | No |
| 83 | RAC1 | na | 21936 | -0.045 | -0.2532 | No |
| 84 | BDNF | na | 22475 | -0.052 | -0.2657 | No |
| 85 | YWHAQ | na | 22775 | -0.056 | -0.2711 | No |
| 86 | ARHGDIB | na | 23266 | -0.064 | -0.2815 | No |
| 87 | CDC42 | na | 23297 | -0.064 | -0.2786 | No |
| 88 | TRAF6 | na | 23526 | -0.068 | -0.2813 | No |
| 89 | CALM3 | na | 23602 | -0.069 | -0.2794 | No |
| 90 | BAD | na | 23795 | -0.072 | -0.2807 | No |
| 91 | ABL1 | na | 23912 | -0.074 | -0.2797 | No |
| 92 | GRB2 | na | 24215 | -0.079 | -0.2838 | No |
| 93 | SHC1 | na | 24263 | -0.080 | -0.2805 | No |
| 94 | NTF3 | na | 24484 | -0.084 | -0.2820 | No |
| 95 | CRK | na | 25398 | -0.101 | -0.3025 | No |
| 96 | NFKBIB | na | 25462 | -0.102 | -0.2983 | No |
| 97 | MAGED1 | na | 25835 | -0.109 | -0.3027 | No |
| 98 | IRAK2 | na | 26241 | -0.118 | -0.3075 | No |
| 99 | MAPKAPK2 | na | 26341 | -0.120 | -0.3033 | No |
| 100 | ARHGDIA | na | 26630 | -0.126 | -0.3043 | No |
| 101 | MAP3K5 | na | 27057 | -0.137 | -0.3086 | No |
| 102 | NGF | na | 27092 | -0.137 | -0.3016 | No |
| 103 | SH2B1 | na | 28226 | -0.167 | -0.3245 | Yes |
| 104 | NFKB1 | na | 28241 | -0.168 | -0.3151 | Yes |
| 105 | RAPGEF1 | na | 28454 | -0.174 | -0.3111 | Yes |
| 106 | CRKL | na | 29092 | -0.194 | -0.3182 | Yes |
| 107 | RPS6KA1 | na | 29168 | -0.196 | -0.3089 | Yes |
| 108 | NTF4 | na | 29548 | -0.208 | -0.3077 | Yes |
| 109 | NRAS | na | 30019 | -0.224 | -0.3082 | Yes |
| 110 | MAP2K1 | na | 30150 | -0.229 | -0.2985 | Yes |
| 111 | RELA | na | 31135 | -0.271 | -0.3112 | Yes |
| 112 | YWHAH | na | 31453 | -0.288 | -0.3034 | Yes |
| 113 | NFKBIA | na | 31525 | -0.292 | -0.2884 | Yes |
| 114 | CAMK2D | na | 31910 | -0.312 | -0.2813 | Yes |
| 115 | SHC4 | na | 32535 | -0.353 | -0.2787 | Yes |
| 116 | CAMK4 | na | 32687 | -0.364 | -0.2618 | Yes |
| 117 | BCL2 | na | 32817 | -0.374 | -0.2437 | Yes |
| 118 | IRS2 | na | 32897 | -0.380 | -0.2238 | Yes |
| 119 | NFKBIE | na | 32930 | -0.382 | -0.2024 | Yes |
| 120 | NTRK2 | na | 33066 | -0.393 | -0.1833 | Yes |
| 121 | RIPK2 | na | 33268 | -0.416 | -0.1648 | Yes |
| 122 | SH2B3 | na | 34398 | -0.632 | -0.1605 | Yes |
| 123 | SHC2 | na | 34640 | -0.800 | -0.1207 | Yes |
| 124 | AKT3 | na | 34696 | -0.932 | -0.0678 | Yes |
| 125 | IRS1 | na | 34720 | -1.176 | 0.0002 | Yes |
Table: GSEA details [plain text format]

  

Fig 2: KEGG\_NEUROTROPHIN\_SIGNALING\_PATHWAY      
 Blue-Pink O' Gram in the Space of the Analyzed GeneSet

  

Fig 3: KEGG\_NEUROTROPHIN\_SIGNALING\_PATHWAY: Random ES distribution      
 Gene set null distribution of ES for **KEGG\_NEUROTROPHIN\_SIGNALING\_PATHWAY**
